# Many hops, many stops: care-seeking “loops” for diabetes and hypertension in three urban informal settlements in the Mumbai Metropolitan Region

**DOI:** 10.3389/fpubh.2023.1257226

**Published:** 2024-01-09

**Authors:** Sudha Ramani, Manjula Bahuguna, Jennifer Spencer, Sweety Pathak, Sushma Shende, Shanti Pantvaidya, Vanessa D’Souza, Anuja Jayaraman

**Affiliations:** Society for Nutrition, Education and Health Action, Mumbai, India

**Keywords:** non-communicable diseases, patient journeys, care-seeking pathways, primary health care, diabetes, hypertension, urban slums, informal settlements

## Abstract

**Background:**

The burden of Non-Communicable Diseases (NCDs) in urban informal settlements across Lower and Middle Income Countries is increasing. In recognition, there has been interest in fine-tuning policies on NCDs to meet the unique needs of people living in these settlements. To inform such policy efforts, we studied the care-seeking journeys of people living in urban informal settlements for two NCDs—diabetes and hypertension. The study was done in the Mumbai Metropolitan Region, India.

**Methods:**

This qualitative study was based on interviews with patients having diabetes and hypertension, supplemented by interactions with the general community, private doctors, and public sector staff. We conducted a total of 47 interviews and 6 Focus Group Discussions. We synthesized data thematically and used the qualitative software NVivo Version 10.3 to aid the process. In this paper, we report on themes that we, as a team, interpreted as striking and policy-relevant features of peoples’ journeys.

**Results:**

People recounted having long and convoluted care-seeking journeys for the two NCDs we studied. There were several delays in diagnosis and treatment initiation. Most people’s first point of contact for medical care were local physicians with a non-allopathic degree, who were not always able to diagnose the two NCDs. People reported seeking care from a multitude of healthcare providers (public and private), and repeatedly switched providers. Their stories often comprised multiple points of diagnosis, re-diagnosis, treatment initiation, and treatment adjustments. Advice from neighbors, friends, and family played an essential role in shaping the care-seeking process. Trade-offs between saving costs and obtaining relief from symptoms were made constantly.

**Conclusion:**

Our paper attempts to bring the voices of people to the forefront of policies on NCDs. People’s convoluted journeys with numerous switches between providers indicate the need for trusted “first-contact” points for NCD care. Integrating care across providers—public and private—in urban informal settlements—can go a long way in streamlining the NCD care-seeking process and making care more affordable for people. Educating the community on NCD prevention, screening, and treatment adherence; and establishing local support mechanisms (such as patient groups) may also help optimize people’s care-seeking pathways.

## 1 Introduction

Recently, urban informal settlements across Low and Middle-Income Countries (LMICs) have noted an increase in the burden of Non-Communicable Diseases (NCDs) (1–3). Evidence suggests that residents of such settlements are at increased risk for NCDs due to a range of factors, including chronic stress, unfavorable working conditions, poor diets, and environmental pollution (4–6). Furthermore, access to high-quality healthcare is frequently lacking in such areas (7, 8). The adverse influence of financial insecurity and social marginalization, added to all of this (9, 10), further compromises NCD outcomes in this population group.

As a consequence, there is interest across LMICs in aligning and fine-tuning health policies to respond to the increasing NCD burden in urban informal settlements. One compelling way to aid such policy endeavors is through empirical understandings of people’s care-seeking journeys for NCDs in these spaces. A care-seeking journey is characterized as a sequence of actions that begins with awareness about something not being right, following which intervention from formal and informal health resources is sought (11). Examining care-seeking journeys can enable contextualized understandings of people’s physical and emotional experiences and, thereby, help include community voices in policy discussions (12–14). Studies of patient journeys in the past have provided policy evidence for strengthening diagnostic processes, tweaking treatment regimens, and reducing delays in the care-seeking process (15, 16). Overall, such inquiries have served as important tools for making health policies more people-centered (17). Indeed, the recent “intent to action” series by the World Health Organization treats the lived experiences of patients as key evidence for improving NCD policies (18).

This study aims to get deeper insights into the care-seeking journeys of people for two NCDs—diabetes and hypertension. The study was done in three informal settlements in the peripheral areas of the Mumbai Metropolitan Region in India. The two NCDs—diabetes and hypertension—were chosen since a high prevalence of these diseases has been reported in urban informal settlements in India (19, 20).

This qualitative study adds to existing evidence on care-seeking journeys for NCDs in LMICs. Earlier studies on this topic have highlighted issues of delayed diagnosis and lack of continuity in NCD care—owing to poor quality primary healthcare systems, cost concerns, and a lack of clarity over where to seek care, *inter alia* (12, 16, 21). We add to this evidence a study that considers the unique context of urban informal settlements, such as the existence of a mixed (public and private) health system and the resource constraints faced by the urban poor in accessing care.

### 1.1 Indian context

In India, Disability-Adjusted Life Years (DALYs) attributed to NCDs has risen from 30% in 1990 to 55% in 2016 (22). A recently undertaken nationally representative population-based study in India estimated the prevalence of diabetes and hypertension as 11.4 and 35.5% respectively, with a higher burden of NCDs in urban areas compared to rural areas (23). Whereas NCDs were earlier perceived as “diseases of the rich” (1), confined to the urban elite, there is increasing recognition that the urban poor are also vulnerable to this set of diseases (24, 25).

The urban public health system, particularly at primary levels of care, is underdeveloped in most places in the country (26, 27). Also, this system has conventionally focussed on providing select services for maternal and child health, and certain infectious diseases of national priority. Though a national program to combat NCDs has existed for many years in the country, the intent to integrate NCD care comprehensively into all tiers of the public health system has only recently gained political priority (28). Presently, policies in India strongly support free screening, treatment, and management of NCDs through the public health system (28, 29). However, the implementation of policies on NCDs has not been without challenges. The addition of NCD services appears to put even more pressure on the nation’s already overburdened and under-resourced public health system (30).

At present, much of primary-level curative care in India happens in the private sector, through out-of-pocket payments, and in the hands of Non Degree Allopathic Practitioners (NDAPs) (31–33). NDAPs practice allopathy without formal qualifications in modern medicine in close proximity to the community (32, 34). There is evidence that NDAPs are often the first-contact points for care for most minor acute ailments in both rural areas and urban slums in the country (32, 35). But at present, their role in the diagnosis and treatment of NCDs is less documented. In general, the nuances of care-seeking for NCDs is less understood in the Indian context. This study is an attempt to elucidate the nuances of care-seeking for two common NCDs, diabetes and hypertension, by the urban poor.

## 2 Methods

### 2.1 Study setting

This study was done in a peripheral Municipal Corporation (urban geographical region under a local governing body) in the Mumbai Metropolitan Region, Maharashtra, India. This corporation has a population of 0.7 million, of which 44% live in informal settlements. The density of people per square kilometer is 26,871 (36). Historically, these settlements grew around a textile industry hub. Hence, many of these settlements are home to migrants who work in the loom industry. The study area has access to a mix of public and private healthcare providers. One public secondary-level hospital (100 beds) serves as a referral unit for 15 primary-level facilities operating under this Municipality.

### 2.2 Study design

This is a cross-sectional, qualitative study that employs quasi-inductive approaches to examine patient journeys. Such approaches allow for context-specific theorization grounded in data from research participants even while pragmatically allowing learnings from existing theories and frameworks to be incorporated in the analysis (37, 38). The data in this study mainly comprises interviews with patients with diabetes (10 patients) or hypertension (9 patients) or both (7 patients) who shared their care-seeking stories with us based on their memories. These interviews were supplemented with information from community-level group discussions (40 participants), visits to the public secondary-level hospital and one primary-level facility in the area (13 participants), and interviews with local private healthcare providers (8 participants). The supplementary information we collected enabled us to understand the care-seeking journeys of patients from a broader perspective that acknowledged the context of healthcare provision in this area.

### 2.3 Participant selection and recruitment

This study was conducted in the field areas of the Society for Nutrition, Education and Health Action (SNEHA), a nongovernmental organization that has been working in urban informal settlements in Mumbai since 1999. SNEHA has a long-standing relationship with the community in the study area. The field staff of SNEHA initially identified patients, community members, and doctors based on the diversity criteria suggested by the research team. In their work related to maternal and child health, the field staff regularly perform home visits within the community. During these visits, they identified individuals within the family who had diabetes and hypertension. They explained to them the purpose of the study and inquired about their willingness to participate. Similarly, SNEHA also works closely with the public health system in the area to establish a stronger link between the healthcare systems and the community. This collaboration allowed us to engage with the public health staff to collect data. The researchers from SNEHA thereafter conducted the interviews and discussions as per the participants’ convenience after a formal process of informed consent. We purposively sampled for diversity in age and gender (all study participants), migration status (all community participants), and years of diagnosis/treatment of the disease (only patients).

### 2.4 Data collection

Data collection was done from September to December 2022. The main topics discussed during our interactions are summarized in Table 1. A total of 34 in-depth interviews (IDIs), 13 short-duration interviews, and 6 Focus Group Discussions (FGDs) were conducted. Out of 26 patients interviewed, 18 were above 50 years of age and many (17) had lived in the community for more than two decades. An equal number of men and women patients (13 each) were interviewed. Diversity in years of diagnosis was ensured—11 patients were diagnosed 2 years prior to the interaction, 7 between 3 and 5 years and 8 were diagnosed more than 5 years prior to the interaction. Forty community members participated in the FGDs, of which 27 were less than 35 years old and 13 were male. Majority (27) had lived in the community for more than a decade. Details of the participants’ demographics are provided in Supplementary Table 1.

**Table 1:** Themes covered during our interactions.

**Main themes**		
**Diabetes and hypertension patients (in-depth interviews)**		
**Story of the patient’s care seeking journey**		
Initial complaints First providers sought The subsequent course of action taken by patients, and providers sought in case of severity or complications The point of diagnosis Adherence-medicines, diet, cost of treatment, reasons for poor adherence Access to healthcare—public and private health providers, relations with healthcare providers, regularity of follow-up Awareness and perceptions of the disease(s)		
**Supplementary themes**		
Diabetes and hypertension patients (supplementary data interviews)	Community members (focus group discussions)	Healthcare providers (interviews)
Reasons for accessing public healthcare Process of accessing healthcare in the public hospital Challenges and limitations in accessing public healthcare	Perceived etiology, perceived symptoms, attitudes toward susceptibility and perceptions of severity of the diseases	Perceptions on diabetes and hypertension in the community Nature of care provided and experiences of treating patients for the two diseases Challenges faced in the provision of care

The participants’ preferred local languages, Hindi and Marathi, were used during the interactions. Authors MB, JS, and SR conducted the interactions; all three are well-trained in qualitative data collection methods and familiar with the local context. On average, the IDIs and FGDs lasted 20–30 min. The interviews with patients were conducted in their homes while those with healthcare professionals were conducted in their workplaces. FGDs, facilitated by a moderator (MB or JS) and a note-taker, were held in anganwadis (local government playschools) or one of the participants’ homes. Short-duration interviews (lasting 10–15 min each) were conducted outside the NCD outpatient department of the public hospital when patients were either waiting for consultation or exiting after consultation. Similar interviews with public health staff were conducted after their working hours on the hospital premises. These interviews took less time since they were meant to provide only supplementary information on patient care pathways, and validate the concerns raised by patients in our study. Such supplementary interviews are a well-established mechanism in qualitative research to triangulate information, in order to ensure data quality (39).

As is the practice in qualitative studies, the exact sample size was not pre-determined. We used the concept of information redundancy as a criterion to ensure data saturation, after which, we stopped the recruitment of additional participants.

### 2.5 Data analysis

All interactions with community members and patients (except the eight short-duration supplementary interviews at the hospital) were recorded. Of the eight interviews with private providers, five were recorded; and of the five interviews with public health staff, three were recorded (as per the participants’ preferences). These recordings were transcribed into English for further analysis. Individuals who were proficient in Hindi, Marathi and English carried out the translation and transcription. Subsequently, MB and JS listened to the audio recordings and reviewed the transcribed content to ensure quality. For the non-recorded interviews, we took detailed field notes. As is typical of qualitative studies, data analysis processes were initiated simultaneously with data collection (40).

We followed the steps that Miles and Huberman recommend for the analysis of data, starting with data reduction (initial condensing), then working with data displays (compressed assemblies of information), and finally, drawing meaningful interpretations (38). We had data debriefing sessions after every data collection visit to discuss emerging themes. Ideas from the transcripts were also discussed in a team to arrive at a standard set of codes. The transcripts and field notes were sorted and coded using the qualitative software NVivo Version 10.3.

### 2.6 The analytical framework used

To choose an analytical framework for our study, we examined existing literature on patient journeys for NCDs. We found that many papers used clinical headings—such as diagnosis, initiation of treatment, follow up and management of the disease—to explicate the patient journey (11, 41–43). For example, a recent review of NCDs in LMICs used the term “touchpoints” (awareness, screening, diagnosis, treatment, and adherence) to describe the patient journey through the health system (11). Other articles have also noted the importance of examining patient journeys through a “continuum of care” approach as care-seeking for NCDs should ideally involve multiple follow-ups with repeated points of contact with the health system (44, 45).

**Figure 1:**
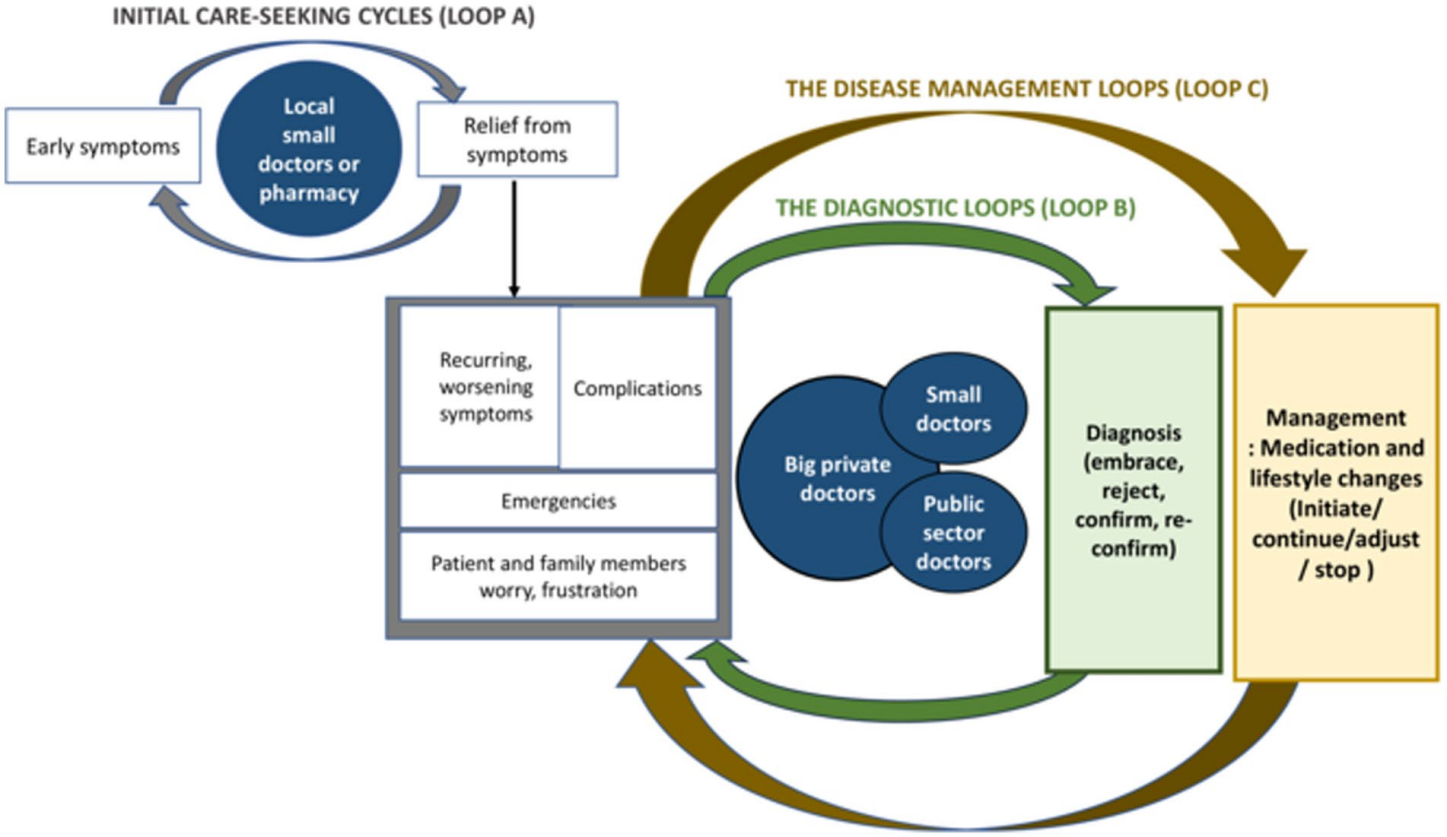
Cyclic care-seeking pathways for diabetes and hypertension (attached separately).

These ideas informed the initial tools of our study. We also based our preliminary analysis on the different “touchpoints” we encountered in patient stories. But our preliminary analysis revealed that some of the unique findings in our context did not fit into the existing frameworks very well. For instance, patient journeys in our study appeared to be highly convoluted and could not be confined to distinct touchpoints alone. Further, given the existence of a mixed (public and private) and pluralistic (allopathic and alternate medicine) healthcare system in India (46), there was constant “hopping” between different providers for NCD care. These issues were not being captured through existing NCD care-seeking frameworks. Thus, in our paper, we have built further on existing ideas and employed quasi-inductive approaches to develop a framework (38) to better represent that chaotic situation of care-seeking for NCDs on the ground. Since this framework evolved iteratively from our data, we have discussed it at the end of the paper (see Figure 1).

### 2.7 Ethical considerations

Ethical approval for the research was obtained from Sigma Research and Consulting Pvt. Ltd. on 28th September 2022. Informed consent was verbally obtained from all participants. In cases where the participant granted permission to audio record the interviews, the consent and ensuing interactions were recorded on a smartphone device. In cases where the participants did not agree to audio recording, non-recorded verbal consent was obtained, and field notes were taken during and after the interaction. Permissions were obtained from the municipal ward corporator responsible for the population under study and relevant public health system authorities in the area.

## 3 Findings

People recounted having long and convoluted care-seeking journeys for the two NCDs we studied. These journeys often involved multiple “hops” between different kinds of providers, as well as experimentation with different types of treatment regimens. In most of the stories we heard, the diagnosis of the two NCDs was delayed until people’s life routines were extensively disrupted due to worsening symptoms. Further, few people spoke of routine follow-ups. In this section, we have described some of the salient features of people’s care-seeking journeys that our team interpreted as noteworthy.

### 3.1 Initial complaints and the first providers sought

Many patients began their stories by talking about symptoms that interfered with their lives. These symptoms often included headaches, body pain, anxiety, dizziness, and tiredness. Since patients’ early memories were hazy, it was challenging to determine how long these symptoms had manifested in people’s lives prior to a formal diagnosis. Most patients said that they dealt with these early, not-very-serious symptoms by going to a nearby source of healthcare, such as a pharmacy (drugstore) for over-the-counter drugs and visiting a local private doctor referred to as a “small” doctor:

*“His clinic is not big but has been there for many years. All members of our family get medicine only from him. On this road, he is a famous doctor. He himself gives the medicine. He takes only fifty rupees” (Female, 32 years, diagnosed with diabetes six months prior to the interaction).*

*“Over time when I started feeling uneasy, I went to my family doctor. He said my BP (blood pressure) was high, so he gave me a pill to keep it on my tongue. He also asked me to visit a big doctor” (Male, 52 years, diagnosed with hypertension eight years prior to the interaction).*

“Small” doctors were typically NDAPs, had private clinics within the narrow lanes of the informal settlements, and were easily accessible to people. Patients shared that “small” doctors spoke kindly, often provided “small” medicines during the consultation, and, importantly, charged fees that they could afford. These fees were typically only a fraction of the fee charged by an urban allopathic doctor (Allopathic doctors charged 500 to 1,000 Indian National Rupees (INR) and in contrast, these doctors took a fee of 50 to 100 INR). The ‘small’ doctors also shared that they occasionally accommodated patients’ requests by deferring taking fees for consultation until the patients could afford to pay them.

Many “small” doctors admitted that they usually provided symptomatic treatment for minor ailments:

*“If someone feels uneasy, I give them medicine to help them relax. I make them lie down with their legs up, and I make them chew on ginger. Because of this, the heaviness in their chest and lack of oxygen starts recovering” (Small doctor, clinical practice for twenty-five years).*

Less than one-fourth of the patients we interviewed had been diagnosed by the “small” doctors. These doctors preferred to refer patients to more qualified doctors for the formal diagnosis and continued treatment of NCDs. Long-term treatment for NCDs was perceived as costly. One doctor also noted that recommending such long-term, costly treatment regimens to patients would undermine their reputation as a reasonably-priced doctor and have a negative impact on their practice.

### 3.2 The subsequent journey involved many “hops” between different kinds of providers

When the symptoms experienced by patients were not controlled as anticipated, people did one or more of the following. They either returned to the “small” doctors with more extensive complaints or reached out to “big” private doctors. “Big” private doctors usually possessed at least an undergraduate degree in allopathic medicine, and their workspaces were outside the informal settlements and sometimes even in adjoining cities. Less often, people reported seeking care from the public sector and non-profit hospitals.

One of the most striking features of this part of the care-seeking journey was the multitude of providers sought by people. Table 2 notes the number of providers sought by each patient we spoke to. We found that most patients switched providers multiple times, including hopping between “small” and “big” doctors, the public and private sectors (profit and non-profit), and the allopathic and alternate medicine sectors. We found only three patients who recalled visiting just one doctor as part of this journey (and two of these three patients reported being diagnosed only 6 months prior to our interaction with them).

**Table 2:** Healthcare providers sought by patients in our study.

Patient number	Sex	Age	Disease	Duration of the disease	Healthcare providers sought									Reasons for switching between healthcare providers
					1	2	3	4	5	6	7	8	9	
1	F	50	D, H	5 years	✓									Confirmation of diagnosis and no relief
2	F	57	D, H	5 years-H	✓									Separate doctor sought for hypertension and diabetes
				1 year-D										
3	F	40	D, H	1 year		✓								No relief
4	F	32	D	6 months	✓									No switch, has stopped medicine due to high cost
5	M	55	D	6 months		✓								No relief
6	F	45	D	6 months	✓									Confirmation of diagnosis before starting medicines
7	M	67	H	2 years	✓									No switch
8	F	45	D	15 years	✓									Finances, migration, low access to medicines
9	F	65	D, H	8 years		✓								No relief and for treatment of co-morbidities, specialist care`
10	F	50	D	12 years	✓									Cost of medicines, medicine did not suit, treatment of co-morbidities
11	F	40	H	3 years	✓									No relief
12	F	55	D	1 year	✓									No relief and high cost of medicine
13	M	75	D	>12 years	✓									Relief, for treatment of co-morbidities
14	M	55	H	4 years	✓									No relief
15	M	53	D	5 years	✓									No relief and treatment of co-morbidities
16	M	86	H	12 years	✓									Migration
17	M	65	D	2 years			✓							No relief
18	M	62	D, H	2 years		✓								For treatment of complications of diabetes
19	F	60	D, H	2 years	✓									No relief, high cost of treatment, treatment of co-morbidities
20	F	35	D	7 years	✓									Switched from obstetrician to general physician, cost of treatment
21	M	56	H	6 years			✓							No relief and treatment of co-morbidities
22	M	55	D, H	4 years	✓									Treatment of co-morbidities and due to the high cost of treatment
23	F	45	H	16 years	✓									Migration, high cost of medicines
24	M	63	H	1 year	✓									Cost of treatment and medicines and treatment of co-morbidities
25	M	66	H	4 years	✓									High cost of treatment
26	M	52	H	8 years	✓									Better care and high cost of treatment
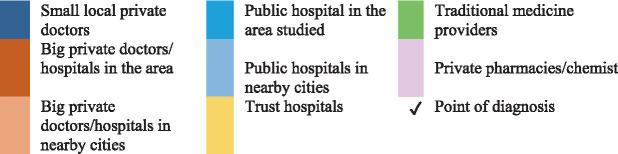														

The care-seeking illustrated in the table focusses on the point of diagnoses and the number of provider switches made after this point. We have not tabulated mentions of their early visits to small providers because most people could not recall the exact numbers of visits and switches made between doctors before their diagnosis. D, Diabetes; M, Male; H, Hypertension. F, Female.

People reported switching providers for multiple reasons, including the confirmation of diagnoses; no improvement in symptoms; in search of better-suited treatment; due to co-morbidities; to avoid the high cost of consultations that people could not afford at certain points in time; and based on the assortment of advice they received from well-wishers on the varied expertise of different healthcare providers.

The reasons shared by people for switching doctors signal the lack of a clear-cut pathway for accessing NCD care. To begin with, the familiar circle of “small” doctors, whom people were accustomed to, could often not diagnose or treat NCDs in this setting. Thus, people had to explore a previously unexplored set of providers—the “big” private doctors—who were more qualified but, at the same time, costlier. Patients often expressed feeling powerless in the face of the high costs they incurred at the “big” private clinics. They also felt uneasy asking the “big” doctors (who were perceived as very busy) for advice and questioned the legitimacy of the tests prescribed by these doctors. This lack of familiarity with the formal private sector also contributed to extensive experimentation with different kinds of providers and diverse treatment regimens. Advice from well-wishers (family and friends) and “try and see” attitudes appeared to guide the entire care-seeking journey. The three cases in Table 3 illustrate some of these points.

**Table 3:** Excerpts from patient stories to illustrate “hopping” between providers.

**Excerpt from Rani’s story**
*Rani was diagnosed with diabetes during her routine antenatal check-ups in a ‘big’ private hospital. She continued taking her diabetes medication up until after her pregnancy, at which point she quit. When she felt unwell again, she went to another private doctor, whose medicines she found to be too expensive to buy on a long-term basis. So, she went to the public hospital (at a distance of 5 kilometers) for free medicine. But after a few months, she found it challenging to go repeatedly to the public hospital for medicine refills due to child-care responsibilities. Hence, she stopped going there. At the time of the interview, she reported going to a small private doctor for check-ups and medicine. But she also added that sometimes she skipped the check-up and took the medicines directly from the local pharmacy (Name changed, female, 35 years, diagnosed with diabetes 16 years prior to the interaction)*.
**Excerpt from Abdul’s story**
*Abdul had a boil on his back, which did not heal for a few days, following which he visited a ‘big’ private doctor, who diagnosed and prescribed medicines for diabetes. When he got typhoid, he went to another doctor for treatment; this doctor changed the diabetes medicine due to his complaints about body itching. But he found this doctor to be rude and expensive, and hence, he never went to him again. At present, he continues the diabetes medicines given to him earlier without follow-up. He sometimes gets his sugar tested on his own and consults yet another small doctor in case of any abnormalities in the results (Name changed, Male, 53 years, diagnosed with diabetes five years prior to the interaction).*
**Excerpt from Lakshya’s story**
*During the COVID-19 lockdown two years ago, Lakshya had trouble breathing. Her family brought her to a nearby camp to undergo testing which did not help. She was admitted to a private hospital as her condition worsened, and following a battery of tests, she was diagnosed with diabetes and hypertension. After her diagnoses, she visited a local private doctor, who gave her diabetic medication. This medicine did not suit her. So, she went to another big private doctor and received a different prescription from him. She only takes diabetes medicine since she cannot afford to take hypertension medication. Six months into her diagnosis, she went to get retested in a free camp organized by a private hospital in an adjoining city, where an angiography and several other tests were done. From this hospital, she received new prescriptions for medication for diabetes and hypertension, and for asthma (diagnosed newly). She also takes medicine from the pharmacy directly as needed, and ayurvedic drugs from a traditional healer (Name changed, Female, 60 years, diagnosed with diabetes and hypertension two years prior to the interaction).*

### 3.3 The care-seeking journey was cyclic, with multiple diagnoses and treatment points

We found that people did not move from awareness to diagnosis and further from diagnosis to treatment in a linear manner. People’s stories often comprised multiple points of diagnosis and re-diagnosis; and multiple points of treatment initiation, adjustment, stopping, and re-initiation. Since these points were often cyclically encountered, we have described these points in terms of two “loops” in the care-seeking journey: the diagnostic loops and the disease management loops. We have explained these two loops below, but also discuss these further in Figure 1 in section 4 of the paper.

#### 3.3.1 The diagnostic loops

Around three-fourths of the patients we interviewed reported that their diagnosis was done at the “big” private doctor (see Table 2, which marks the point of first diagnosis). However, this diagnosis did not immediately or always lead to effective treatment and management. This was because patients often ignored the early diagnosis, only to be re-diagnosed subsequently after the symptoms worsened. A few went to a range of doctors to confirm their diagnosis before believing it. For instance, one woman who was diagnosed with diabetes by a skin specialist found it hard to see the connection between diabetes and her skin problems, and struggled to believe her diagnosis:

*“I had a cut that did not heal and became painful. I went to a private skin doctor, who diagnosed me with diabetes. But my family was worried and asked me to confirm. I went to another doctor to confirm, and there it also showed up. I went to five doctors in total. When they all told me I have sugar (diabetes), that is when I believed it” (Female, 60 years, diagnosed with diabetes five years prior to the interaction).*

Some others got diagnosed, initiated, and then stopped treatment and subsequently had to be re-diagnosed. Additionally, the re-diagnosis was frequently done by healthcare providers who were not necessarily aware of the patient’s history.

There were two departures from this typical diagnostic pathway. One departure occurred when the diagnosis happened incidentally. We interviewed five patients who had gone to the hospital for various reasons (such as a COVID-19 test or vaccine or a pregnancy check-up) and discovered that they had diabetes or hypertension by chance. The second departure occurred in five cases, where the diagnosis was made extremely late, when the patients were admitted to a hospital, frequently with a stroke or a heart attack. The quote below is from one such patient:

*“I had a heart attack, and I went from one private hospital to another before finally going to a public hospital in Mumbai. There, they did my angioplasty… At that time, I found out that I had hypertension” (Male, 63 years, diagnosed with hypertension two years prior to the interaction).*

#### 3.3.2 The many loops in the management of diabetes and hypertension

We found several variations in the ways that patients worked with their treatment regimens. They sometimes completely stopped all medicines; or adjusted dosages on their own; or took partial medicines; or reserved costlier allopathic medicines only for emergencies while opting for home remedies and alternative medicine on a routine basis. In many stories, people reported taking medication in a cyclic manner. That is, they stopped when they felt better or had financial constraints; and restarted when the symptoms emerged again. Fatima’s case below is an illustrative example of how people adjusted their medicine cyclically.

*Fatima lived in a modest one-room house with her family. She had a bedridden husband and a daughter who was mentally challenged. Her son earns, and she supplements his income by pearl-beading necklaces. Upon being diagnosed with diabetes, she took medicines from a private doctor (who charged her reduced fees). Fatima took these medicines for some time, but when she started feeling better, she stopped taking them to save money. After a few months, her symptoms reappeared. She again went to the doctor, who requested that she continue the medicine. However, the cost of medicine was too high for her. So, she went to the public hospital for free medicines on the advice of a neighbour. But she felt the medicines given by the public hospital were too strong for her, and she discontinued them. Currently, she purchases the medicines prescribed by the earlier private doctor whenever she has some money and stops them on days when she feels better (Name changed, female, 55 years, diagnosed with diabetes one year prior to the interaction).*

A few patients also reported restarting their discontinued medicines after facing dire consequences (like a heart attack). Such patients often admitted to having “learnt their lesson” on playing with medication. Adherence to treatment also appeared to benefit from the presence of supportive family members. In this regard, male patients frequently referred to their wives, who would remind them to take their medications and make wholesome meals for them to eat.

### 3.4 The constant balancing act between getting relief and lowering costs

Throughout their care-seeking journeys, people in urban informal settlements continually attempted to strike a compromise between the need for relief and the need for lowering costs. For instance, when poor health hindered livelihoods and daily routines, patients shifted from “small” private doctors to more expensive big private doctors. Conversely, when people felt better, and the symptoms were less severe, or the costs of big private doctors became burdensome, they shifted from private to public doctors and from costlier allopathic medicines to cheaper traditional medicines. People also tried to save costs by adjusting their treatment regimens—they took medicines intermittently, bought partial medicines, or continued medicines without regular check-ups. The following quotes emphatically illustrate the trade-offs that people made between saving money and getting relief.

*“I am not able to take medicines regularly. The diabetes medicine I was advised, they are so expensive. If we have money, I get it (medicines). Otherwise, I take one Ayurvedic syrup. Only if my condition gets worse, I take the allopathy medicine” (Female, 60 years, diagnosed with diabetes two years prior to the interaction).*

*“Now I am old, so they (the loom owners) do not use me for running the machines. I am sitting idle for one and a half months with no work. If I go to the private doctor, he will give me two injections and three-times medicine and will take 100 rupees. If he writes medicine (to be bought) from outside, then 150 rupees. He gave me a strip of medicine from which I take half a tablet every day (Male, 65 years, diagnosed with diabetes two years prior to the interaction).*

*The doctor lectures me and says that when he tells me first to get checked up and then take the medicine, I do not listen to him. To save a few rupees, I buy the medicine from the shop and take it (Female, 45 years, diagnosed with hypertension 16 years prior to the interaction).*

Recognizing the inability of vulnerable population groups to pay out of pocket for chronic illnesses, free services for hypertension and diabetes has been made available in the public health sector in India. However, these free services were perceived as being far from adequate by the community. Interviews at the referral hospital in the area revealed that though the facility had a specific cell for NCDs, the doctor’s position has been vacant for the last 6 months. When we visited, an overworked substitute doctor was overseeing the diagnosis and the disbursal of medicines for a few NCDs. Also, we were told during the interviews that medicines for both diabetes and hypertension were not always available or sufficient. The public sector outreach workers associated with primary-level public health facilities were only partially trained and had not yet formally been assigned responsibilities pertaining to NCD care. Further, we found that the community was often unaware of the availability of NCD services in the public sector. People also shared past experiences of long waiting hours, rudeness of staff, and feelings of not being treated well in public facilities in the area studied. Quoting from one patient interview:

*“It is not that we do not want to go there (public hospital). We would save some hard-earned money if we do that. If only they provide us with information properly” (Female, 32 years, diagnosed with diabetes six months prior to the interaction).*

Such experiences generally discouraged care-seeking from the public health sector or compelled patients to travel to better public hospitals in nearby cities in the metropolitan region when needed.

### 3.5 Lay perceptions: notable absence of the concept of prevention of NCDs in communities

#### 3.5.1 Perceptions of the community about diabetes and hypertension

In our FGDs with the community, we found that people were generally aware of both diabetes and hypertension. Women seemed to know more details about testing for diabetes and hypertension than men due to the checkups done during pregnancy. We present below the perceptions of the community under four themes in brief—perceived etiology, perceived symptoms, attitudes toward susceptibility and perceptions of severity. Table 4 depicts illustrative quotes from each of the themes below.

Perceived etiology: Poor diet and stress were perceived as triggers of both diabetes and hypertension. Heredity and other lifestyle issues (lack of exercise, obesity) were not mentioned by participants as contributors. Religious beliefs also shaped perceptions of the causes and impact of the diseases.
Perceived symptoms: People shared that diabetes manifests as weakness in the body, aches and pains, wounds that do not heal, frequent urination and dizziness, and hypertension manifests as dizziness, anxiety, confusion and anger.
Attitudes toward susceptibility: Most people believed that the young are not susceptible. Old age and tension were seen as precipitators. Both men and women were seen as equally susceptible to diabetes and hypertension.
Perceptions of severity: There was consensus that both conditions were dangerous and disruptive to people’s lives in many ways. Patients particularly noted diet restrictions and the challenges in adhering to these restrictions if diagnosed with diabetes or hypertension.

**Table 4:** Community perceptions on diabetes and hypertension.

Perceptions of the community	Sample quotes
Perceived etiology	*There is a lot of work at home, and if you do not pay attention to your food and drink, you can get BP (hypertension) (Female, FGD 18–40 years age group).*
	*When you sin, you get stressed and your health gets worse (Male, 67 years, diagnosed with hypertension 2 years prior to the interaction).*
Perceived symptoms	*I have heard that in sugar (diabetes), if there is any cut, it will not heal, the person gets weak, the eyes get weak, and the person gets thin. It is of two types; in one person gets hungry and, in another person does not get hungry (Female, FGD 30–60 years age group).*
Attitudes toward susceptibility	*In young blood, you usually do not see BP (hypertension), sugar (diabetes). When a person gets weak, in old age, or has tension, then these things happen (Male, FGD 18–40 years age group).*
Perceptions of severity	*BP (hypertension) is dangerous because in BP you feel dizzy and you cannot do any work, you cannot function, and the body becomes loose (Female, FGD 18–40 years age group).*
	*Everything you cannot eat (in diabetes), whatever you like to eat you cannot eat. If you have to control too much, it is as good as dying of hungry (Female, FGD 18–40 years age group).*
Attitudes toward screening	*No, we have not gone (for the screening test). We are poor, we do not have money to just go to do such tests without cause. Now we do not have anything (symptom) then why would we go for testing? (Male, FGD 18–60 years age group).*

#### 3.5.2 Influence of lay perceptions on people’s care seeking journeys

The above perceptions of diabetes and hypertension influenced people’s care-seeking journeys in many ways. For one, these perceptions did not engender the need for preventive lifestyle changes or early screening. It was believed that stressful events caused the body to be weak and precipitated such ailments. Since stressful and emotional life events were perceived as beyond the control of participants, notions of preventing diabetes and hypertension were largely missing from people’s narratives. While patients recognized the dire consequences of the diseases, they felt the need to take action only if these disrupted daily routines and ability to work. Therefore, screening which involved precious costs in terms of time and money, was considered illogical in the absence of bothersome symptoms.

While people acknowledged the role of diet control as necessary in managing diabetes and hypertension after diagnosis, changing lifestyles and diets as preventive measures were not mentioned in the general community. Several patients/family members spoke of practicing diet restrictions, while others said it was challenging due to the lack of availability of healthy food where they worked. But we did not find any patients who emphasized the importance of exercise in managing these two NCDs. When we probed on this, two patients mentioned the lack of space near their houses to walk or exercise. Others regarded their daily work as adequate exercise. As one female participant in a community discussion told us, *“We have so much work in the household, we are on our feet, bending-whether it is filling water or washing, that is our exercise.”*

## 4 Discussion

We have structured our discussion in two sections. The first section discusses the study’s contribution to conceptual thinking on care-seeking for NCDs in LMICs. The second section summarizes the key policy implications of this study.

### 4.1 Care-seeking pathways for NCDs in LMICs

Figure 1 is our attempt to represent the diverse care-seeking pathways for NCD care encountered in our study in the form of a framework. Figure 1 depicts three cyclic components of the pathway: the initial care-seeking cycles (loop A), the diagnostic loops (loop B), and the disease-management loops (loop C). Loop A comprises the pursuit of symptomatic relief from “small” private doctors and local pharmacies. People usually exit this loop only when their symptoms worsen, making it challenging for them to carry on with routine activities. On exiting loop A, people enter the diagnostic loops (loop B). Here, people embrace, confirm, or reject diagnoses. Diagnosis is often not a single point in the patient journey but occurs repeatedly in a loop-like fashion. Intersecting with the diagnostic loops are the disease management loops (loop C). In loop C, people initiate, adjust, stop, or restart disease management. In both loops B and C, care is sought from a mix of healthcare providers.

Our framework attempts to add conceptual richness to current thinking on care-seeking pathways for NCDs. It acknowledges that care-seeking journeys for NCDs can be messy and cyclic in low-resource settings; and cannot be linearly portrayed in simplistic ways. While it includes the two touchpoints-diagnosis and treatment used in earlier studies (11, 43, 44), these get considered as cyclic occurrences (“loops”) in our framework rather than as sequential events. Further, the framework explicitly depicts “hopping” providers, a phenomenon that is of particular concern in LMICs.

It can be argued that cycles are a positive phenomenon in NCD care-seeking and that the occurrence of multiple diagnostic and disease management cycles signal “continuum of care” for these ailments (45, 46). But in our data, repeats in diagnosis or changes in the course of treatment, were typically not a part of meticulous “follow-up” by patients. Instead, these repeats were most often a response to previously disregarded diagnoses or poorly followed treatment advice.

Our study found that people’s care seeking pathways were characterized by numerous hops between diverse providers. The reasons that people reported in our study for hopping providers—such as costs, distances, quality of care, and patient satisfaction—echo findings from other informal settlements in India, Kenya, and other African contexts (47–49). But, in a broader sense, the sheer number of “hops” in almost every patient journey clearly signal constant dissatisfaction with the care that people received. The “hops” are indicative of the urgent need for trusted and reliable “first-contact” points for NCD care, as well as for the integration of care provided by different providers—two essential characteristics of good primary care (50).

The phenomenon of “hopping” providers in our study also explains why the bypass of primary care, an important issue of policy concern (51, 52), is challenging to comprehend in LMICs. Our data suggests that, with respect to care for NCDs, people do not “bypass” one provider in favor of another. Instead, they experiment with multiple options for care, sometimes even simultaneously, and continually search for options that balance the need for relief and the need to save on healthcare costs.

Our findings also point to delays in diagnosis and problems with treatment adherence. Diagnostic delays in our study had many causes, including the lack of providers who advised on necessary screening tests during the early stage of the disease and people’s resistance to believing the diagnosis. We felt that there were many missed opportunities for early detection, an issue of concern in other LMIC settings as well (16, 43, 53). With regard to adherence to treatment for hypertension and diabetes, our findings were consistent with those reported in other LMICs, and included both structural factors (cost of medicines and lack of continuity of care) and behavioral factors (lack of knowledge and unfavorable attitudes toward long-term treatments) (54–56). Similar to another study in urban India, we found that the lack of adherence to treatment regimens was intentional and did not stem from mere forgetfulness of patients (57). In summary, though patients gave a variety of explanations for issues in diagnosis and treatment, these issue stemmed from the absence of affordable and integrated healthcare in our study area.

### 4.2 Policy implications

We have summarized the key learnings from this paper in Table 5. From our findings, we suggest below three ways in which NCD care for people living in urban informal settlements in India can be strengthened.

**Table 5:** Summarized learnings from the study.

Conceptual learnings	Policy learnings
New thinking on care-seeking pathways for NCDs must acknowledge the messiness of this process, and not shy away from accounting for non-linear, cyclic events in patient journeys. In contexts like ours, frameworks to capture patient experiences must consider the plurality of providers available and sought. A less complicated journey for patients should be a goal for policies on NCDs to work toward.	The public sector in urban areas needs to be strengthened to adequately screen, diagnose, and treat diabetes and hypertension. We particularly advocate for outreach screening and follow-up in urban slums. Non-degree allopathic practitioners are the ubiquitously available “first-contact” points for healthcare in urban slums. Their presence and strong networks can be better leveraged to facilitate routine screening, diagnosis, and management of NCDs. Raising awareness in the community is an important step in optimizing the care-seeking pathway for NCDs.

First, the urban public health sector needs to play a more active and integrated role in managing NCDs. Recent studies show that the public health sector in India is not being utilized adequately for the screening, treatment, and follow-up of NCDs (58, 59). One recent survey confirms that, despite recent policy efforts, the preparedness of India’s public primary and secondary care facilities to integrate care provision for NCDs is weak (60). Also, the public health system in most of India at present lacks the strong coordination and follow-up mechanisms needed to effectively treat chronic diseases (61, 62). While the integration of NCDs into primary care has begun in many places in the country, more concerted action on this front is needed, particularly in urban areas.

Second, the government-run NCD programs in the country’s urban areas can benefit from better engagement with the “small” doctors (NDAPs). The advantages of “small” doctors in delivering first-contact primary care, such as their intimate ties to the community, responsiveness to the needs felt by the community, and affordability, have been extensively discussed in literature (63, 64). Concerns have also been raised regarding the quality of care that “small” doctors offer (65). Despite these concerns, small providers have been leveraged by the National Tuberculosis Control Program (66) and recently during the COVID-19 pandemic (67) to strengthen service delivery. In terms of NCDs, the role of these doctors has so far been limited. Since these doctors are often the first points of healthcare contact for the community, their non-involvement speaks to a missed opportunity for screening and early diagnosis of NCDs. Training this set of healthcare providers on NCD screening and diagnosis and leveraging their community networks to support the public sector in NCD care can go a long way in streamlining the care-seeking journey for people. Across LMICs, the plurality of providers in urban health systems is being increasingly recognized (68). While it is imperative to strengthen the urban public sector for NCD care, the role that the local private sector in such areas can play cannot be ignored.

Lastly, advice from neighbors and friends, as well as support from families, plays an important role in shaping people’s care-seeking journeys. We found that while the community was aware of diabetes and hypertension as diseases, there was a paucity of knowledge about how the diseases were linked to lifestyle and heredity. Notions of “prevention” and “screening” for NCDs were entirely missing from community narratives. Raising awareness in the community is an important first step to optimizing the care-seeking pathway for NCDs. For doing so, “educational” interventions such as group communication, individual counseling, and mass awareness campaigns—including interventions led by community health workers and lay facilitators—have been suggested (69). Experiences from LMICs also tout patient “support” groups as a valuable strategy for empowering patients, improving long-term adherence to medicines, and spreading awareness about NCDs (70, 71).

While this study offers fresh perspectives by analyzing patient care seeking journeys for non-communicable diseases in urban informal settlements, it has some limitations. One limitation arises from gathering information through memory recall. Some patients could remember only key events in their care-seeking journeys, such as the treatment during worsening symptoms or catastrophic health events. Many could not remember dates and concrete timelines. Often, patients did not relate several “every day” symptoms to their disease, providing little information about their journeys before diagnosis or about simultaneous journeys related to other diseases. We tried our best to deal with recall bias and specifically probe and clarify events. While we tried our best to diversify the participants involved in our study, we could not completely eliminate selection bias. Since the patients were sampled through SNEHA’s existing maternal and child health program in the community, the selection of participants included only those with families living in the informal settlements. We also did not have access to recently migrated male workers who were single and living in this area, and we acknowledge that their care-seeking journeys could have been different.

### 4.3 Conclusion

This study found that care-seeking for NCDs in urban informal settlements was convoluted, with patients hopping from one provider to another. People in our study area reported experimenting with three healthcare options—the local private sector comprising of “small” doctors, the formal allopathic private sector, and the public sector. The first option—the “small” doctors were regarded by the community as both affordable and accessible; however, these doctors could not always diagnose and treat NCDs. The second option—the formal private sector, comprising providers trained in allopathic medicine, was regarded by this population as both costly and unfamiliar. The third option—the urban public health sector was often considered as being difficult to access, inadequate in terms of coverage, and non-sympathetic to people’s felt needs. Thus, none of the three healthcare options seemed to meet the NCD care needs of people living in the urban informal settlements we studied.

Empirical studies of care-seeking journeys like ours can serve as important tools for policies to understand people’s needs and expectations. The findings of this study suggest that there is an urgent need for trusted “first-contact” points for NCD care and for integrating care across healthcare providers in urban informal settlements. Raising awareness in the community is also an important step in optimizing the care-seeking pathway for NCDs. Urban health policies should strive to make journeys less challenging for patients, rather than assuming that care-seeking events linearly unfold from awareness to diagnosis and treatment.

## Data availability statement

The original contributions presented in the study are included in the article/Supplementary material, further inquiries can be directed to the corresponding author.

## Author contributions

SR: Conceptualization, Supervision, Data curation, Formal Analysis, Methodology, Writing – original draft. MB: Conceptualization, Data curation, Formal Analysis, Methodology, Writing – original draft, Project administration. JS: Data curation, Formal Analysis, Project administration, Writing – original draft, Methodology. SwP: Project administration, Conceptualization, Writing – review & editing. SS: Conceptualization, Writing – review & editing, Supervision. ShP: Conceptualization, Supervision, Writing – review & editing, Validation. VD’S: Supervision, Writing – review & editing. AJ: Supervision, Writing – review & editing, Conceptualization, Funding acquisition, Validation, Methodology, Project administration, Resources.
