# Supplementary material for: Many hops, many stops: care-seeking “loops” for diabetes and hypertension in three urban informal settlements in the Mumbai Metropolitan Region
Source: Front Public Health. 2024 Jan 9;11:1257226. doi: 10.3389/fpubh.2023.1257226 (PMC10803512; doi:10.3389/fpubh.2023.1257226)
Supplement: Supplementary file 1 [file Table_1.DOCX]

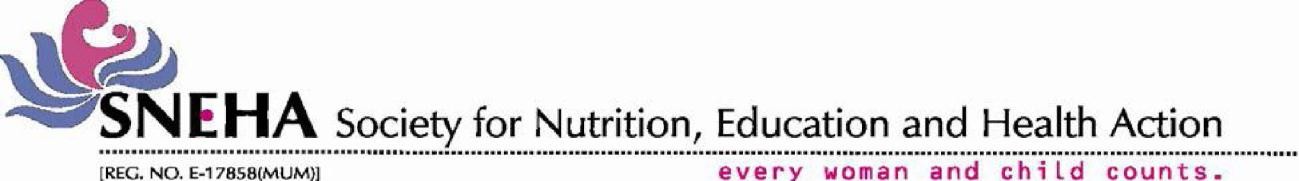


# **Focus group discussion guide- Community awareness, attitudes and health-seeking towards diabetes and hypertension**

**Gender of the group:**

| **No** | **Age** | **Religion** | **Marital status** | **Employment** | **Years of residence in the community/ migrant** |
| --- | --- | --- | --- | --- | --- |
| **1** |  |  |  |  |  |
| **2** |  |  |  |  |  |
| **3** |  |  |  |  |  |
| **4** |  |  |  |  |  |
| **5** |  |  |  |  |  |
| **6** |  |  |  |  |  |
| **7** |  |  |  |  |  |
| **8** |  |  |  |  |  |
| **9** |  |  |  |  |  |
| **10** |  |  |  |  |  |

**Introduction to the discussion**

Hello! Thank you so much for taking out the time and participating in this discussion. I am ………………and these are my colleagues ……………. and ……………...from SNEHA. Today, we are conducting this group discussion to mainly talk about two diseases- diabetes and hypertension. We would like to know about your views related to these two diseases and about the health services available for the diagnosis and treatment of diabetes and hypertension in this community. We would like to understand if there are any challenges in seeking care for diabetes and hypertension in this community.

There are no right or wrong answers to these questions, so please feel free to share your views and experiences with us. At any point, if you think you do not understand any questions or you do not want to participate any further, please let us know. Before we start, if anybody has any questions, you can please ask now.

****Bullets are probing questions to be used if needed***

| **Today we all be talking about some diseases like sugar (diabetes) and blood pressure (hypertension).** | |
| --- | --- |
| **Diabetes** | |
| **Awareness about diabetes** | 1.So, in this community, have you heard about sugar ki bimari? Can you please share what do you know?  2 In the opinion of people here, what are some of the things that happen to one’s body if one has sugar ki bimari? |
| **Screening** | 3. When does one feel the need to test blood for Sugar?   - Should one check for sugar even when someone is feeling completely alright? (should everyone check? Should people over a certain age check? Should pregnant women check?)  1. Has anyone ever given you advise on getting screened? (Probes- who? Has anyone from the public health system advised you to get screened? Any private doctor? Anganwadi worker?) |
| **Diagnosis** | 1. How does one come to know one has sugar ki bimari?  - Where does one do such tests? - How did you come to know about centers mentioned above for diagnosis?  1. Do you know people who have gone for such tests? Do they face any issues or challenges in diagnosis? |
| **Perceived severity** | In your opinion, would you consider sugar ki bimari as an important problem in this community? Why and why not? |
| **Care-seeking for sugar in the community** | 1. In this community, who are the usual doctors people go to when they fall sick?   Besides taking medicines, are there other things people do to control sugar? What are these? |
| **Care seeking stories** | Could you tell us some stories of people with Sugar ki bhimari? |
| ***As we discussed about sugar ki bimari, now we will talk about blood pressure problem in the community.*** | |
| **Hypertension** | |
| **Awareness about hypertension** | 1. So, have you heard about blood pressure problem? Can you please share what do you know?   In your opinion, what are some of the things that happen to one’s body if one has blood pressure problem? (Probe for knowledge of different symptoms) |
| **Screening** | 1. When does one feel the need to test for blood pressure?  - Should one check for blood pressure even when someone is feeling completely alright? (Should everyone check? Should people over a certain age check? Should pregnant women check?)   Has anyone ever given you advise on getting screened? (Probes- who? Has anyone from the public health system advised you to get screened? Any private doctor? Anganwadi worker?) |
| **Diagnosis** | 1. How does one come to know one has blood pressure problem?  - Where can one measure their blood pressure? - How did you come to know about centers mentioned above for diagnosis?  1. Could you tell us if there are any difficulties in getting BP diagnosis done in this community? |
| **Perceived severity** | In your opinion, would you consider the blood pressure problem as an important problem in this community? Why and why not? |
| **Care-seeking for hypertension in the community** | 1. In this community, where one does go for blood pressure problem? Are these the same doctors you mentioned for sugar? (Probe for all public, private, AYUSH, informal, laboratories, chemist shops, NGOs, etc.)  - Why do people go to this facility/doctor?  1. Besides taking medicines, are there other things people do to control blood pressure? What are these? (Probes- Is there any difference in care seeking by age and gender) |
| **Care seeking stories** | Could you tell us some stories of people with BP problems? |
| **Generic questions-comparing NCDs and others** | Is there any difference between seeking care for a small ailment (short duration-cough cough fever and sugar/BP? If so, what would these be? |


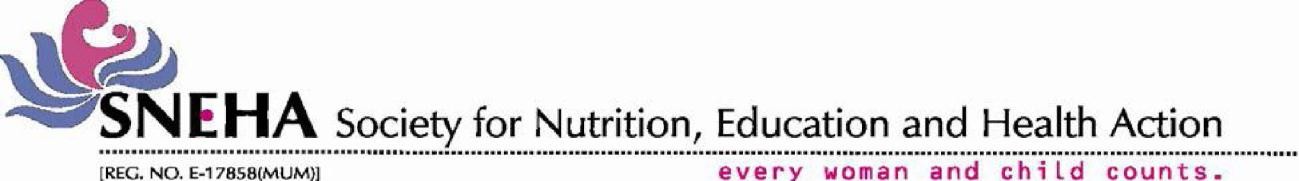


# **In-depth interview guide- Care-seeking journeys of patients with diabetes and hypertension**

**Basic demographic profile of the participant**

| Age |  |
| --- | --- |
| Gender |  |
| Religion |  |
| Marital status |  |
| Employment |  |
| Years of residence in the community/Migrant |  |
| Family details |  |

****Bullets are probing questions to be used as needed***

| **Hello, we would like to talk about your experiences in dealing with sugar/blood pressure.** | |
| --- | --- |
| **Awareness of symptoms before diagnosis** | 1. Can you tell me from the beginning the story of when and how you came to know you had diabetes/Blood pressure?   - When you got diagnosed, did you know anything about sugar/blood pressure? Did you have any symptoms? - Did you try something to control your symptoms? (Probes- preventive measures, home remedies) - Did you talk to somebody about your issues? What advise did they give? (Probes- home remedies, traditional practitioners) |
| **Decision to seek care** | 2. Could you tell us a little about how you decided about seeking care for the bimari?   - When did you decide to go see a doctor for symptoms you were experiencing? - Did you ask somebody where to go? Who suggested where to go? - Can you tell me about your first visit to that facility/doctor? What did the doctor say to you? (Probes- Did the doctor explain the diseases in detail? Did the doctor talk about the risk factors and advise some preventive measures?) |
| **Diagnosis** | 3. Could you elaborate for us the story of how you got diagnosed?   - Did the same facility/doctor do your sugar/blood pressure test or were you sent somewhere else? - Did you show your report to some other doctor? Why? |
| **Treatment** | 4. Could you tell us more details about your treatment?   - Did you start medicines immediately? (Probe for any delays between confirmed diagnosis and initiation of treatment) Was there any issue/worries? - Did you try something else for treating sugar/blood pressure? (Probes- misconceptions, home remedies, traditional practitioners) |
| **Treatment adherence** | 5. You have to take medicines day-after-day for this condition? Could you tell us a little about your treatment experience?   - Do you face any other issues in with the treatment? - At present, are you doing regular testing of sugar/blood pressure? How frequently do you check your sugar/blood pressure? Do you face any difficulty in doing regular check-ups? - Are they any other things you do to manage sugar/blood pressure? What are some of these? |


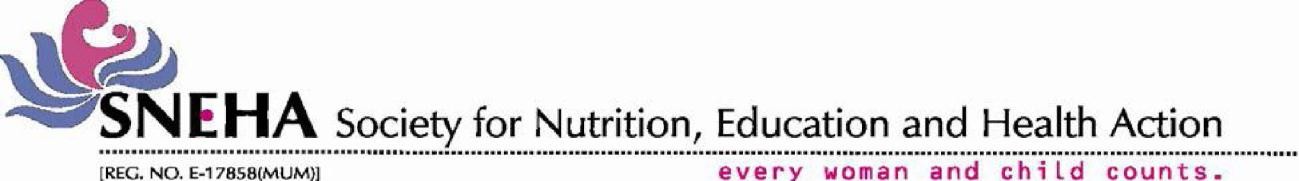


# **In-depth interview guide- Role of public health systems in providing care for diabetes and hypertension**

**Basic demographic profile of the participant**

| Age |  |
| --- | --- |
| Gender |  |
| Designation |  |
| Number of years in service |  |
| Name of the facility |  |
| Number of years in the facility |  |

| **General questions** | 1. In other studies, we have read, we have seen an increase in diabetes and hypertension in recent years. Has this been your experience in this community? |
| --- | --- |
| **Services for diabetes and hypertension**  ***Community screening*** | 2. Are there any specific services that your facility provides for diabetes and hypertension?   - Can you share some stories where you advised a person to get screened for diabetes or hypertension?   3. Do people come for diabetes and hypertension screening in the community? |
| ***Diagnosis and treatment*** | 4. Could you tell us a little about the facilities in this hospital for diagnosis and treatment?   - In cases of multiple diseases or complications, what do you do? Can you manage them in this facility or do you refer them to a higher facility? Can you share any stories where you provided treatment to such cases? - Do you face any difficulty in diagnosing or treating patients with diabetes/hypertension? What are some of the main challenges you face? |
| **Community attitude towards diabetes and hypertension** | 5. In your opinion, what do people think about diabetes in this community? (Probe- Do they consider it dangerous?)  6. What about hypertension, what do people think about hypertension in this community. (Probe- Do they consider it dangerous?)   - When do people usually visit you? (Checking- Do they come for regular check-ups themselves? Or only when they experience any symptoms? Do they come with the onset of symptoms or when their condition gets worse?) |
| **Challenges to access care and treatment for diabetes and hypertension** | 7. Besides giving medicines, do you get time to advice on other issues to patients (eg diet)  8. In your opinion, what challenges do people usually face in accessing treatment for diabetes and hypertension in this community? |


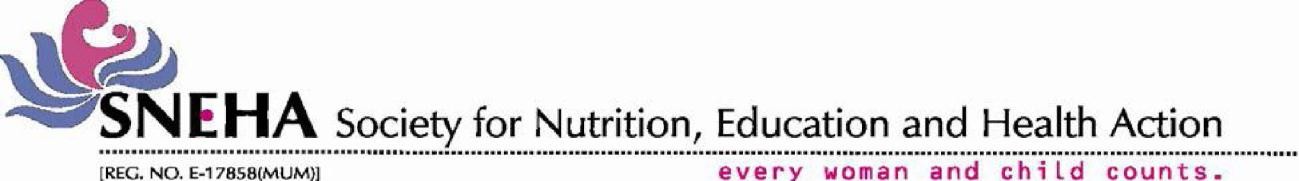


# **In-depth interview guide- Role of private health care providers in providing care for diabetes and hypertension**

**Basic demographic profile of the participant**

| Age |  |
| --- | --- |
| Gender |  |
| Degree/Diploma |  |
| Number of years in clinical practice |  |
| Type/Name of the facility |  |
| Number of years working in the facility |  |

| **General questions** | 1.In other studies, we have read, we have seen an increase in diabetes and hypertension in recent years. Has this been your experience in this community?   - Have you come across patients with diabetes ar hypertension in this community? - Are there any specific services that you provide for diabetes and hypertension? (Probes- screening in the community, testing facility for diabetes and hypertension, treatment)   2. In your opinion, where do people usually go to for diagnosis and treatment of these conditions? |
| --- | --- |
| **Services for diabetes and hypertension** | 3.Could you tell us a little about the facilities in this clinic for diagnosis and treatment?   - In cases of patients with multiple diseases or complications, what do you do? Can you manage them in your facility or do you refer them to a higher facility? Can you share any stories where you provided treatment to such cases? - Do you face any difficulty in diagnosing or treating patients with diabetes/hypertension? What are some of the main challenges you face? |
| **Community attitude towards diabetes and hypertension** | 4.In your opinion, what do people think about diabetes in this community? (Probe- Do they consider it dangerous?)   - When do people generally come to see you? (Probe- Do they come for regular screening on their own? or only when they experience any symptoms? Do they come with the onset of symptoms or when their condition worsens?)   5.What about hypertension, what do people think about hypertension in this community. (Probe- Do they consider it dangerous?)   - Besides the medicines, what advice do you usually give to people with high sugar and blood pressure? (Probe- prevention and management advice) |
| **Challenges to access care and treatment for diabetes and hypertension** | 6.In your opinion, what challenges do people usually face in accessing treatment for diabetes and hypertension in this community?  7.In your opinion, what would help to make diabetes and hypertension care better in this community? |
