# Supplementary material for: Many hops, many stops: care-seeking “loops” for diabetes and hypertension in three urban informal settlements in the Mumbai Metropolitan Region
Source: Front Public Health. 2024 Jan 9;11:1257226. doi: 10.3389/fpubh.2023.1257226 (PMC10803512; doi:10.3389/fpubh.2023.1257226)
Supplement: Supplementary file 2 [file Table_2.DOCX]

**Table 1: Details of Study Participants**

| **Participants** | **Patients of diabetes and hypertension** | | | | | **Community** | **Private doctors** | **Public health staff** |
| --- | --- | --- | --- | --- | --- | --- | --- | --- |
| *Methods of data collection* | *In-depth interviews (26)* | | | | *Short interviews (public hospital) (8)* | *Focus Group Discussion (6)* | *In-depth interviews(8)* | *Short interviews(5)* |
|  | Diabetes | Hypertension | Both | Total |  |  |  |  |
| **Total number of participants (87)** | 10 | 9 | 7 | 26 | 8 | 40 | 8 | 5 |
| **Age (years)** | | | | | | | | |
| <35 | 1 | 0 | 0 | 1 |  | 27 | - | - |
| 35-50 | 4 | 2 | 1 | 7 |  | 10 | - | - |
| 51-65 | 4 | 4 | 6 | 14 |  | 3 | - | - |
| >65 | 1 | 3 | 0 | 4 |  | 0 | - | - |
| **Gender** | | | | | | | | |
| Male | 4 | 7 | 2 | 13 |  | 13 | 7 | 2 |
| Female | 6 | 2 | 5 | 13 |  | 27 | 1 | 3 |
| **Years of Residence in the community** | | | | | | | | |
| <10 | 2 | 1 | 0 | 3 |  | 13 | 0 | - |
| 10 to 20 | 2 | 4 | 0 | 6 |  | 15 | 4 | - |
| >20 | 6 | 4 | 7 | 17 |  | 12 | 4 | - |
| **Employment** | | | | | | | | |
| Employed | 5 | 5 | 2 | 12 |  | 21 | 8 | 5 |
| Unemployed/Retired | 5 | 4 | 5 | 14 |  | 19 | 0 | 0 |
